# Colorectal adenocarcinoma in Uganda: are right-sided and left-sided colon cancers two distinct disease entities?

**DOI:** 10.1186/s12957-023-03094-7

**Published:** 2023-07-22

**Authors:** Richard Wismayer, Julius Kiwanuka, Henry Wabinga, Michael Odida

**Affiliations:** 1 Department of Surgery, Masaka Regional Referral Hospital, Masaka, Uganda; 2 Department of Surgery, Habib Medical School, IUIU University, Kampala, Uganda; 3 Department of Pathology, School of Biomedical Sciences, College of Health Sciences, Makerere University, Kampala, Uganda; 4 Department of Epidemiology and Biostatistics, School of Public Health, College of Health Sciences, Makerere University, Kampala, Uganda; 5 Department of Pathology, Faculty of Medicine, Gulu University, Gulu, Uganda

**Keywords:** Colorectal adenocarcinoma, Stage, Grade, Lymphovascular invasion (LVI), Right-sided colon cancer, Left-sided colon cancer

## Abstract

**Introduction:**

In Western countries, right-sided colon cancers (RSCC) present at an older age and advanced stage. Researchers believe that there is a difference between left-sided colon cancer (LSCC) and RSCC. In Uganda, however, it is unknown whether differences exist in the pathological profile between RSCC and LSCC. The aim of this study was to determine the differences in clinicopathological characteristics between RSCC and LSCC in Ugandan patients.

**Methodology:**

A cross-sectional study was conducted in which colorectal adenocarcinoma formalin-fixed paraffin-embedded tissue (FFPE) blocks were obtained from 2008 to 2021. Colorectal specimens were obtained from prospectively recruited patients. In the retrospective study arm, FFPE blocks and data were obtained from the archives of pathology laboratory repositories. Parameters studied included age, sex, location of the tumour, grade, stage, lymphovascular (LVI) status, and histopathological subtype between LSCC and RSCC.

**Results:**

Patients with RSCC were not older than those with LSCC (mean age, 56.3 years vs 53.5 years; *p* = 0.170). There was no difference in the stage between RSCC and LSCC. Poorly differentiated tumours were more commonly found in RSCC than in LSCC (18.7% vs 10.1%; *p* = 0.038). Moderately and poorly differentiated colonic tumours were more common with RSCC (89.3%) than with LSCC (75.1%) (*p* = 0.007). Younger patients had more poorly differentiated tumours than older patients (19.6% versus 8.6%; *p* = 0.002). LVI was more common with RSCC than with LSCC (96.8% vs 85.3%; *p* = 0.014). Mucinous adenocarcinoma (MAC) was more common with RSCC (15.8%) compared with LSCC (8.5%) (*p* = 0.056) although statistical significance was borderline.

**Conclusions:**

Clinicopathological features of RSCCs tend to be different from those of LSCCs. RSCCs tend to be associated with MAC, a higher grade and LVI status compared to LSCC. LSCC and RSCC present predominantly with an advanced stage; therefore, national screening programmes for the early detection of CRC are necessary to reduce mortality in our Ugandan population.

**Supplementary Information:**

The online version contains supplementary material available at 10.1186/s12957-023-03094-7.

## Introduction

Colorectal cancer (CRC) is the third most prevalent type of cancer, accounting for nearly 2 million new cases each year, and is the second leading cause of cancer-related deaths globally [1, 2]. In terms of incidence, CRC is the third most common malignancy in men and the second most common malignancy in women worldwide [2]. In 2040, the global cancer burden is anticipated to reach 28.4 million cases, and developing low-income countries are anticipated to register 64–95% of all these cases [2]. The increase in CRC burden in these countries may be due to the adoption of risk factors such as increased consumption of alcohol, smoking, and a Westernized diet and partly due to an ageing population [3, 4].

During the last 30 years, there has been increased interest regarding the distribution of CRC in the different segments of the rectum and colon in East Africa [5]. An increased incidence of right-sided colon cancers (RSCC) has been reported in the Western world [5–9]. In the Western developed world, there is speculation that more RSCCs have been diagnosed with the widespread use of colonoscopy [10, 11]. However, in rural regions of East Africa, access to colonoscopy is not readily available.

Differences between left-sided colon cancer (LSCC) and right-sided colon cancer (RSCC) have been found in their clinical presentation and gross pathology [10–12]. LSCCs tend to present with large bowel obstruction due to constricting and infiltrating lesions. RSCC presents with iron-deficiency anaemia and is polypoid in nature. Furthermore, differences in the molecular biology between RSCC and LSCC were found in several studies [13–18]. Compared to rectal cancers and LSCCs, RSCCs have more frequent EGFR pathway aberrant activation, higher rates of BRAF and PIK3CA mutation rates and an increased rate of other mutations. Rectal cancers have been found to have higher rates of TOP01 expression and Her2/neu amplification than colonic tumours.

These molecular differences may be responsible for the differences in clinical presentation between LSCC and RSCCs. In Western developed high-income countries, differences between LSCC and RSCC have been found in the pathology profile and staging which are the most important prognostic factors following curative resection for colorectal cancer. RSCCs present at a higher stage and grade and are more aggressive. Furthermore, the mucinous adenocarcinoma (MAC) and signet ring colorectal carcinoma (SRCC) histopathological subtypes, which are associated with a higher stage and grade, are more common in RSCC in Western developed countries. These pathological features in RSCC are more aggressive and are associated with a poorer prognosis.

In Uganda, the question remains whether differences in the pathology profile and staging exist between RSCC and LSCC. The objective of this study was to determine possible differences in the clinicopathological characteristics between RSCC and LSCC in an indigenous population in East Africa.

## Methodology

This was a descriptive cross-sectional study carried out on CRC tissue specimens obtained from colorectal cancer patients. The prospective arm involved colorectal cancer participants recruited from the Department of Surgery of Masaka Regional Referral Hospital, Mulago National Referral Hospital, Uganda Martyr’s Hospital Lubaga and Mengo Hospital. Patient data and CRC tissue specimens were also obtained retrospectively as formalin-fixed paraffin-embedded tissue (FFPE) blocks from the archives of the Department of Pathology, School of Biomedical Sciences, College of Health Sciences, Makerere University and Multisystems Histology Laboratory, Kampala, Uganda. This Department of Pathology at Makerere University receives colorectal cancer tissues from hospitals in different regions of Uganda.

Biopsies and resected colorectal tissue specimens were obtained as FFPE tissue blocks from September 2019 to September 2021 from prospectively recruited colorectal cancer patients while the archived FFPE tissue blocks were obtained from January 2008 to August 2019 for the retrospective arm of the study. Histologically diagnosed colorectal adenocarcinoma samples were included. Colorectal adenocarcinoma samples taken from patients after having had chemotherapy or radiotherapy treatment, poor-quality tissue block samples, duplicate samples, tissue samples with incomplete or unavailable data and CRC tissue blocks lacking demographic data were also excluded.

For all tissue samples, we extracted data on the following variables using a standard data extraction form: age, sex, location of the primary tumour, nodal stage and presence or absence of metastasis. A radiological stage was used and classified according to the TNM AJCC 8th edition for all patients studied [19].

Participants with cancer of the transverse colon, hepatic flexure, ascending colon and caecum were considered to have RSCC [20]. LSCCs included participants with cancer of the rectum, rectosigmoid, sigmoid colon, descending colon and splenic flexure [20].

The histopathological subtypes were classified as classical adenocarcinoma (AC), mucinous adenocarcinoma (MAC) and signet ring colorectal carcinoma (SRCC). The WHO Pathologic classification of colorectal adenocarcinoma was used to classify the histopathologic subtypes [21]. The grade and lymphovascular (LVI) status were obtained by two consultant pathologists on haematoxylin and eosin staining. AC was defined as having classical glandular formation and configured glandular structures. MAC was defined as having large glandular structures with pools of extracellular mucin with more than 50% of the tumour occupied by extracellular mucin. SRCC was defined by the presence of > 50% of tumour cells having signet ring cell features and having an intracytoplasmic mucin vacuole that pushes the nucleus to the periphery [21]. This histological grade of colorectal carcinoma was determined using the WHO classification system: well-differentiated (G1), moderately differentiated (G2) or poorly differentiated (G3) depending on the extent of glandular appearance [21]. Adenocarcinomas displaying more than 95% gland formation were considered grade I, grade 2 in those between 50 and 95% gland formation and grade 3 in those with less than 50% gland formation [21]. The presence of LVI was denoted by 1, and the absence of LVI was denoted by 0 [21]. Two consultant pathologists carried out these laboratory investigations at the Department of Pathology, School of Biomedical Sciences, College of Health Sciences, Makerere University.

## Statistical analysis

We calculated the median and interquartile range (IQR) for age, while categorical variables were summarized as frequencies and percentages and presented as tables and graphs. The distribution of RSCC and LSCCs was obtained and presented by selected patients’ clinicopathological characteristics. Differences in the distribution of RSCC and LSCC by selected clinicopathological characteristics were determined using the Pearson chi-square test for proportions, while differences in the mean age were determined using Student’s *t*-tests. Among the RSCCs, deviation from a linear trend for age was determined using the Cochran-Armitage trend test. In all statistical tests, a *p*-value ≤ 0.05 was considered significant.

## Results

Of the 404 patients included in this analysis, 200 (49.5%) were males, 259 (64.1%) had a colorectal resection and 145 (35.9%) had a biopsy during proctosigmoidoscopy or colonoscopy.

**Fig. 1:**
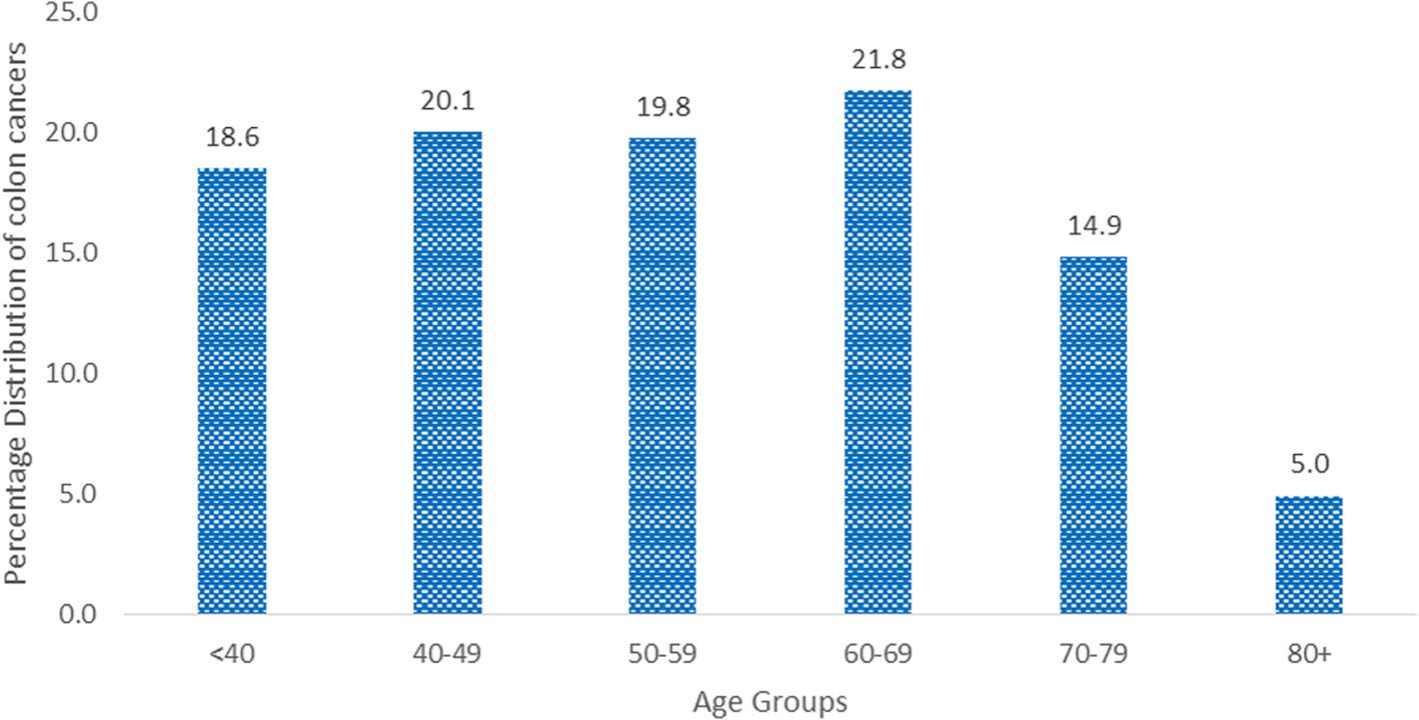
Bar chart showing the age distribution of colon tumours among all the patients

The median (IQR) age of all the patients was 54 (43–67) years, and 38.9% were ≤ 49 years of age (Table 1 and Fig. 1). LSCCs were predominant in all age groups, ranging from 84.0% in patients < 40 years to 70.0% among those aged ≥ 80 years. Similarly, LSCC was 83.0% and 78.9% among males and females, respectively. Fourteen (70.0%) LSCCs and 6 (30.0%) RSCCs were 80 + years of age (Table 2). The mean age (SD) at diagnosis was higher among female patients (55.6) (16.6) than among male patients (52.4) (15.4) (*p* = 0.042).

**Table 1:** Demographic data for all recruited participants

Characteristic	No. of subjects	Per cent (%)
Variable	*N* (404)	
Median age (IQR) (years)	54 (43–67)	
< 40 years	75	18.6
40–49 years	81	20.1
50–59 years	80	19.8
60–69 years	88	21.8
70–79 years	60	14.9
≥ 80 years	20	5.0
Gender (%)		
Male	200	49.5
Female	204	50.5

**Table 2:** Demographic characteristics of the studied group of CRC participants

Characteristics	Left-sided colon, *n* (%)	Right-sided colon, *n* (%)
Age		
< 40	63 (84.0)	12 (16.0)
40–49	66 (81.5)	15 (18.5)
50–59	69 (86.3)	11 (13.7)
60–69	70 (79.6)	18 (20.4)
70–79	45 (75.0)	15 (25.0)
80 +	14 (70.0)	6 (30.0)
Male	166 (83.0)	34 (17.0)
Female	161 (78.9)	43 (21.1)

### Demographics of patients with RSCC versus LSCC

The mean age (SD) for RSCC was 56.3 (16.6) years, while for LSCC, it was 53.5 (16.0) years, and the difference was not statistically significant (*p* = 0.170). In RSCC, the proportion of women was higher than in patients with LSCC (43/77, 55.8% vs 161/327, 49.2%); however, this difference did not reach statistical significance (*p* = 0.297).

Among patients 60 years or older, the proportion with RSCCs was greater with increased age compared to LSCCs. RSCCs compared to LSCCs were 23.4% versus 21.4% for 60–69 years, 19.5% versus 13.8% for 70–79 years and 7.8% versus 4.3% for 80 + years.

**Fig. 2:**
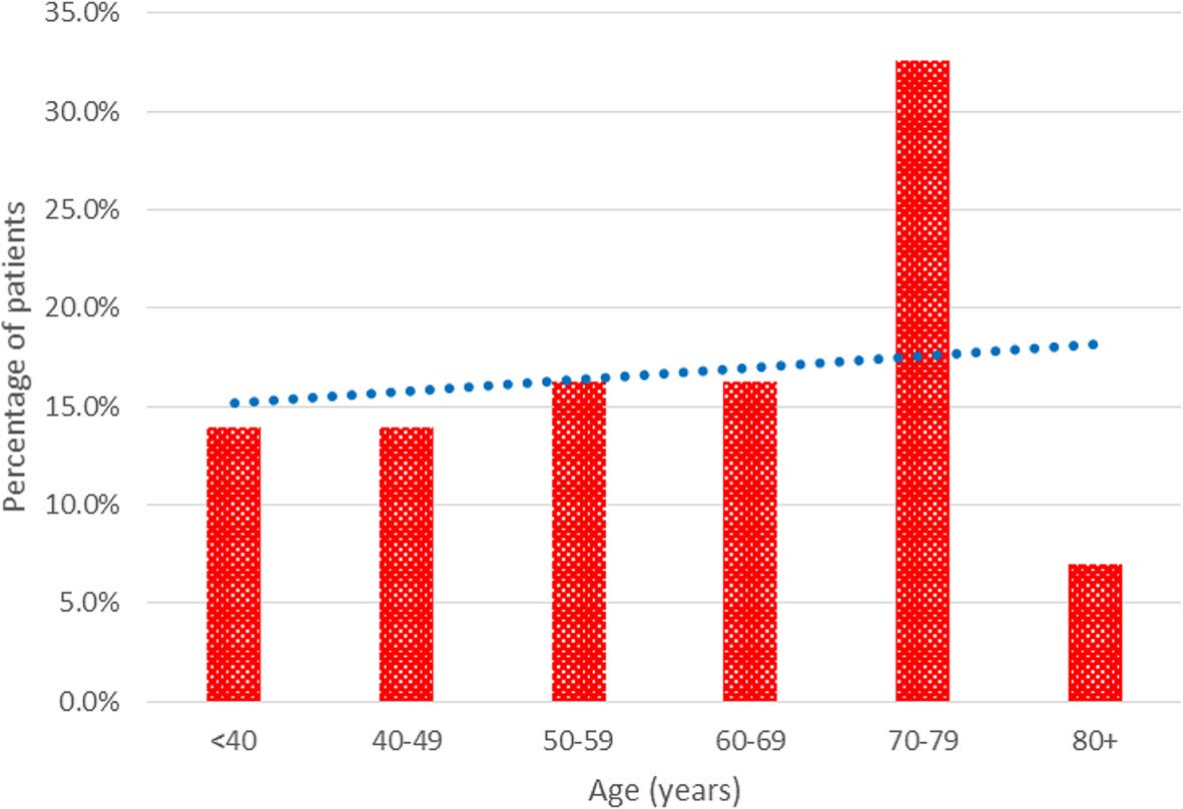
Percentage of female patients with RSCCs by age. *Cochran-Armitage chi-square for departure from linear trend (*p* = 0.128)

**Fig. 3:**
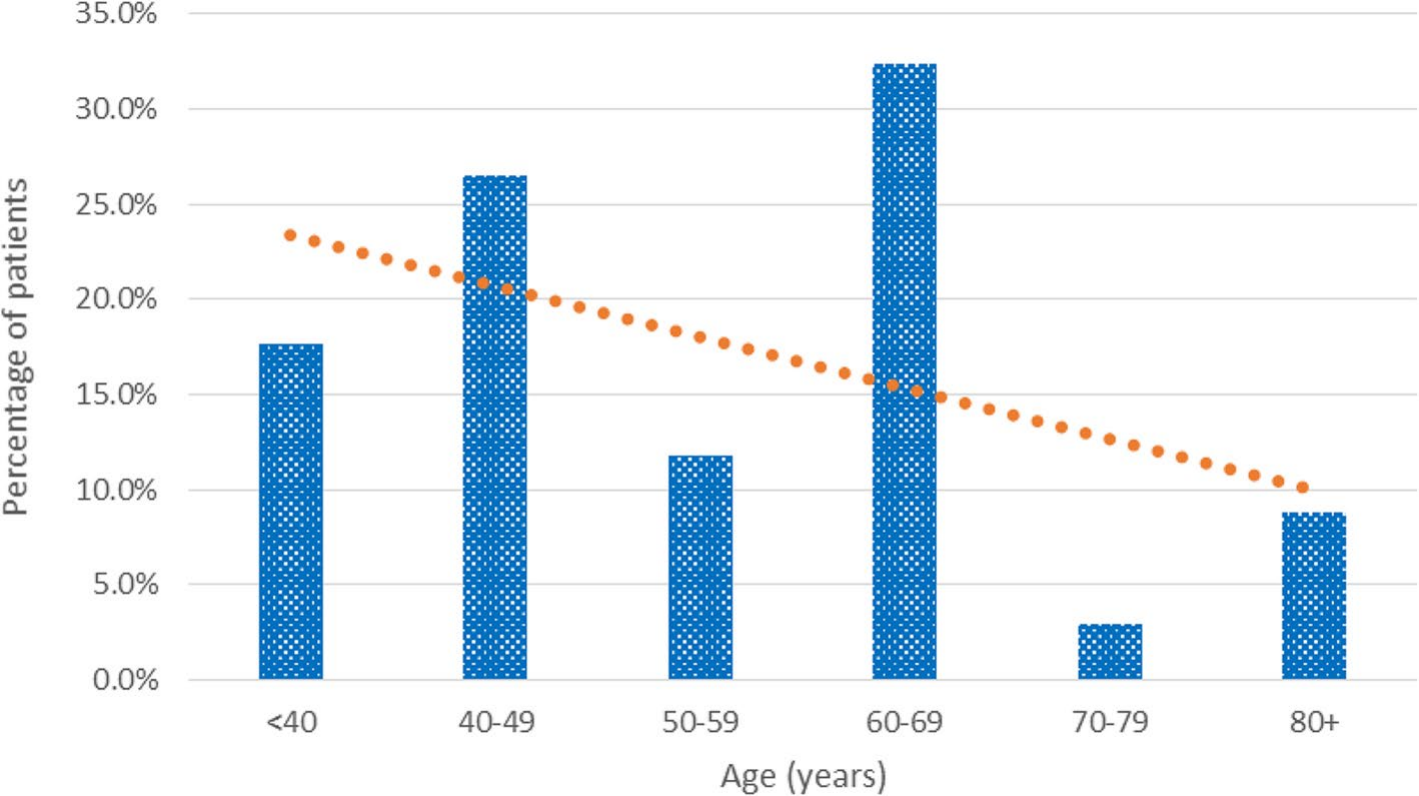
Percentage of male patients with RSCCs by age. *Cochran-Armitage chi-square for departure from linear trend (*p* = 0.534)

Figures 2 and 3 show the proportions of female and male patients with RSCCs, respectively. The proportion of females with RSCCs increased with age, while the proportion of male patients with RSCCs decreased with age. Across genders, the Cochran-Armitage trend test indicated a linear trend (*p* > 0.05).

**Fig. 4:**
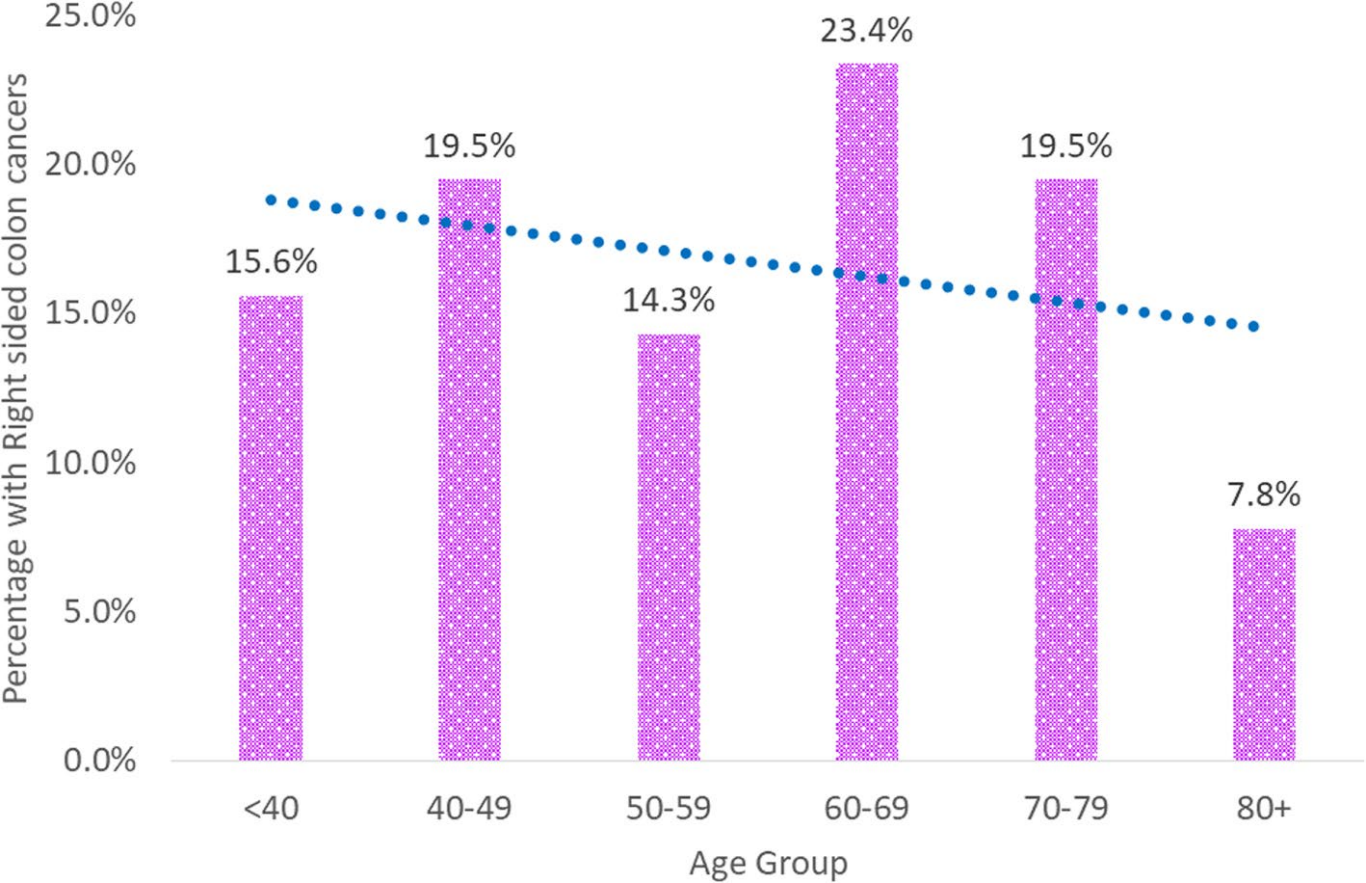
Percentage of all patients with RSCC

However, for all patients, the proportion of RSCCs decreased with age and further exhibited a linear trend (Cochran-Armitage trend *p* = 0.088) (Fig. 4).

Compared to males of the same age group, the proportion of female patients aged 70–79 years was higher (22.1% versus 7.5%) (*p* < 0.001). The proportion of female patients aged 80 + years among all colon cancers was comparable to that of males (5.9% versus 4%) (*p* = 0.384) (Table 3).

**Table 3:** The comparison between the proportions of male and female patients in all age groups

	Male	Female	*p*-value
< 40	40 (20)	35 (17.2)	0.463
40–49	43 (21.5)	38 (18.6)	0.471
50–59	46 (23.0)	34 (16.7)	0.110
60–69	48 (24.0)	40 (19.6)	0.285
70–79	15 (7.5)	45 (22.1)	< 0.001
80 +	8 (4.0)	12 (5.9)	0.384

The proportion of female patients in the 70–79-year-old age group was higher (22.1% versus 7.5%), and this difference reached statistical significance (*p* < 0.001). While the proportion of female patients in all the colon cancers in the 80 + age group was high (5.9% versus 4%), this did not reach statistical significance (*p* = 0.384) (Table 3).

### Topography of colorectal tumours

**Fig. 5:**
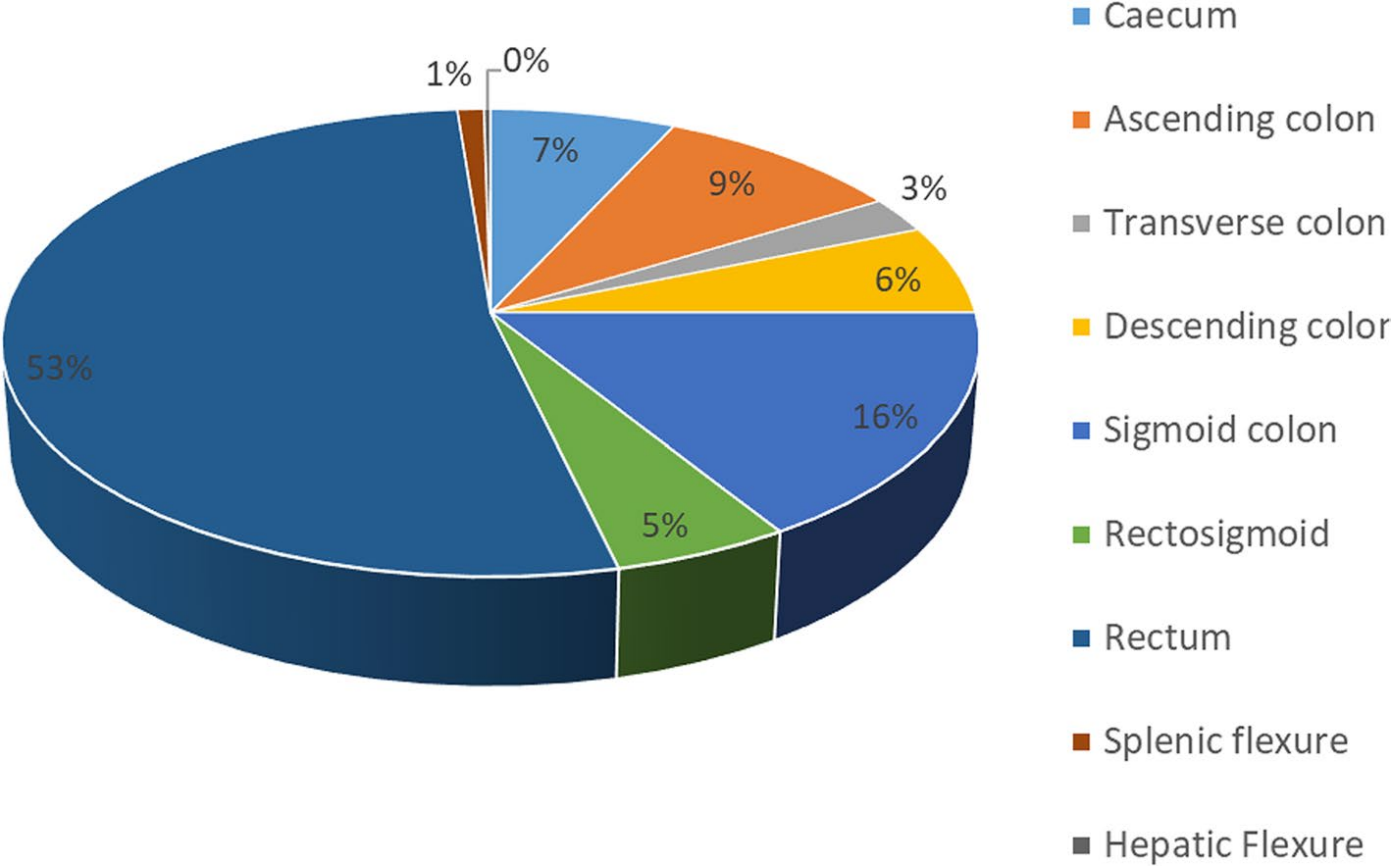
Pie chart showing the anatomical distribution of all colorectal tumours

There were 327 (80.9%) LSCCs, while 77 (19.1%) were RSCCs (Table 4). Overall, rectal tumours were the most common 212 (53%), followed by sigmoid colon tumours 65 (16%) and the least were hepatic flexure tumours 1 (0.3%) (Fig. 5).

**Table 4:** Anatomical distribution of all RSCC and LSCC patients

Location	Number (%)
Right colon	*N* = 77
Caecum	28 (36.4)
Ascending colon	38 (49.4)
Transverse colon	10 (13.0)
Hepatic flexure	1 (1.3)
Left colon	*N* = 327
Descending colon	25 (7.7)
Sigmoid colon	65 (19.9)
Rectum	212 (64.8)
Rectosigmoid	21 (6.4)
Splenic flexure	4 (1.2)

Similarly, among LSCCs, the majority 212 (64.8%) were rectal tumours, followed by 65 (19.9%) sigmoid colon tumours, and the fewest were splenic flexure tumours 4 (1.2%). For RSCCs, 38 (49.4%) were ascending colon tumours, 28 (36.4%) were caecum tumours, and 1 (1.3%) was a hepatic flexure tumour. There were no differences in tumour locations between male and female patients (Table 5). With regard to age, there was also no difference in tumour location between young (≤ 45 years) and older (≥ 46 years) patients apart from tumours in the transverse colon (RSCCs), which were more common in young patients (*p* = 0.026) (Table 6).

**Table 5:** Tumour site differences between males and females

Site	Males, *N* (%)	Female, *N* (%)	*p*-value
Caecum	13 (6.6)	15 (7.5)	0.728
Ascending colon	14 (7.1)	23 (11.9)	0.098
Transverse colon	7 (3.5)	3 (1.5)	0.190
Descending colon	14 (7.1)	11 (5.7)	0.510
Sigmoid colon	35 (17.7)	30 (14.9)	0.457
Rectosigmoid	9 (4.6)	12 (6.0)	0.525
Rectum	106 (53.5)	106 (52.7)	0.873

**Table 6:** Tumour site differences between young (≤ 45) and older (46 +) patients

Site	Younger (≤ 45 years), *N* (%)	Older (≥ 46 years), *N* (%)	*p*-value
Caecum	6 (5.3)	22 (7.7)	0.385
Ascending colon	9 (7.9)	29 (10.2)	0.481
Transverse colon	6 (5.3)	4 (1.4)	0.026
Descending colon	7 (6.1)	18 (6.3)	0.947
Sigmoid colon	17 (14.9)	48 (16.8)	0.637
Rectosigmoid	7 (6.1)	14 (4.9)	0.619
Rectum	62 (54.4)	150 (52.6)	0.750

### Clinicopathological characteristics between RSCC versus LSCCs

Table 7 shows that there was no difference between LSCC and RSCC in male and female patients (LSCC: male 166 (50.8) versus female 161 (49.2%); RSCC: male 34 (44.2%) versus female 43 (55.8%) (*p* = 0.297)).

**Table 7:** Clinicopathological differences between RSCC and LSCC

Characteristic	Left-sided colon (*n* = 327) (%)	Right-sided colon (*n* = 77) (%)	*p*-value
Sex			
Male	166 (50.8)	34 (44.2)	0.297
Female	161 (49.2)	43 (55.8)	0.297
T1	13 (4.6)	3 (4.2)	0.907
T2	47 (16.4)	5 (7.0)	0.045
T3	110 (38.5)	29 (40.9)	0.712
T4	116 (40.56)	34 (47.9)	0.263
T3, T4	226 (79.0)	63 (88.7)	0.062
Lymph node status			
No	105 (42.3)	19 (31.7)	0.131
N1	97 (39.1)	27 (45.0)	0.404
N2 + N3	46 (18.6)	14 (23.3)	0.402
Metastasis			
Mo	242 (84.6)	56 (78.9)	0.243
M1	44 (15.4)	15 (21.1)	0.243
Stage			
Stage I	48 (16.8)	8 (11.3)	0.253
Stage II	70 (24.5)	14 (19.7)	0.397
Stage III	116 (40.6)	30 (42.3)	0.795
Stage IV	52 (18.2)	19 (26.8)	0.105
Grade (%)			
G1 (well differentiated)	79 (24.9)	8 (10.7)	0.008
G2 (moderately differentiated)	206 (65.0)	53 (70.7)	0.349
G3 (poor differentiated)	32 (10.1)	14 (18.7)	0.038
G2 + G3 (moderate or poorly differentiated)	238 (75.1)	67 (89.3)	0.007
Lymphovascular invasion (LVI)	203 (85.3)	60 (96.8)	0.014
Histological subtype			
SRCC	13 (4.1)	3 (4.0)	0.956
MAC	27 (8.5)	12 (15.8)	0.056
AC	278 (87.4)	61 (80.3)	0.106

Comparing the depth of cancer invasion revealed that LSCCs present at an earlier stage (T2) than RSCCs (16.4% versus 7%), and this difference reached statistical significance (*p* = 0.045). Advanced stage (T3, T4) tumours were more commonly found with RSCCs than with LSCCs (88.7% versus 79%), and this nearly reached statistical significance (*p* = 0.062) (Table 7).

Poorly differentiated (G3) tumours were more commonly found in RSCCs than in LSCCs (18.7% versus 10.1%), and this difference reached statistical significance (*p* = 0.038). Moderately and poorly differentiated (G2 + G3) colonic tumours were more common with RSCCs than with LSCCs (89.3% versus 75.1%; *p* = 0.007) (Table 7). Overall, more patients with well-differentiated histology (G1) presented with LSCCs (24.9%) than with RSCCs (10.7%), and this difference reached statistical significance (*p* = 0.008). Younger patients (< 45 years) had more poorly differentiated G3 tumours than older patients (≥ 46 years), and this difference reached statistical significance (22 (19.6%) versus 24 (8.6%); *p* = 0.002).

The presence of lymphovascular invasion (LVI) was more common in RSCCs than in LSCCs (96.8% versus 85.3%), and this difference reached statistical significance (*p* = 0.014) (Table 7).

Histological examination revealed that MAC was more common in RSCCs than in LSCCs (15.8% versus 8.5%), and this difference reached borderline statistical significance (*p* = 0.056). There were no differences in the distribution of MAC between younger (≤ 45 years) and older (≥ 46 years) patients (*p* = 0.258). Thirteen (4.1%) patients had SRCC in the RSCC group, and 3 (4.0%) had SRCC in the LSCC group, which was not statistically significant (*p* = 0.956) (Table 7).

Table 8 shows the distribution of cancer sites by grade and stage. For stage I–III patients, LSCC and RSCCs were comparably distributed in each of the grades (in all, *p* > 0.05). For stage IV and grade I, more patients had LSCCs than RSCCs (*p* = 0.007), but the distribution of right- and left-sided colon cancers was comparable for grade 2 and 3 patients. However, there were more advanced (stage III and stage IV) G3 RSCCs (21.3%) than LSCCs (11.7%), although this difference was not statistically significant (*p* = 0.092). A similar distribution of LSCC and RSCCs was observed among patients in grades 1 and 2, when the stage was categorized as not advanced disease. For grade 3 patients, there were more RSCCs (18.2%, 4/22) than LSCCs (6.2%, 7/113), although the statistical significance was borderline (*p* = 0.060).

**Table 8:** Grade distribution according to the stage of CRC

Stage	Location	G1	G2	G3
I	Rt. colon (*n* = 8)	1 (12.5)	5 (62.5)	2 (25.0)
	Lt. colon (*n* = 48)	11 (22.9)	33 (68.8)	4 (8.3)
	*p*-value	0.506	0.726	0.158
II	Rt. colon (*n* = 14)	1 (7.1)	11 (78.6)	2 (14.3)
	Lt. colon (*n* = 65)	14 (21.5)	48 (73.9)	3 (4.6)
	*p*-value	0.213	0.713	0.178
III	Rt. colon (*n* = 29)	4 (13.8)	19 (65.5)	6 (20.7)
	Lt. colon (*n* = 112)	30 (26.8)	68 (60.7)	14 (12.5)
	*p*-value	0.145	0.635	0.260
IV	Rt. colon (*n* = 18)	0 (0.0)	14 (77.8)	4 (22.2)
	Lt. colon (*n* = 51)	16 (31.4)	30 (58.8)	5 (9.8)
	*p*-value	0.007	0.150	0.179
Not advanced disease (stages 0, I, II)	Rt. colon (*n* = 22)	2 (9.1)	16 (72.7)	4 (18.2)
	Lt. colon (*n* = 113)	25 (22.1)	81 (71.7)	7 (6.2)
	*p*-value	0.162	0.920	0.060
Advanced disease (stages III, IV)	Rt. colon (*n* = 47)	4 (8.5)	33 (70.2)	10 (21.3)
	Lt. colon (*n* = 163)	46 (70.2)	98 (60.1)	19 (11.7)
	*p*-value	0.005	0.208	0.092

For stage III and IV (advanced disease) and grade I patients, more LSCCs (28.2%, 46/163) than RSCCs (8.5%, 4/47) were observed (*p* = 0.005). However, there were more patients with advanced stage (III, IV) disease presenting with G2 and G3 RSCCs than with LSCCs, although this difference did not reach statistical significance (G2: RSCC: 33/47: 70.2% vs LSCC: 98/163: 60.1%; *p* = 0.208; G3: RSCC: 10/47: 21.3% vs LSCC: 19/163: 11.7%; *p* = 0.092).

For stages I, II and III, there was a poorer pathology profile for RSCCs. However, in none of these stages did this trend reach statistical significance. However, when early disease (stages 0, I, II) of RSCC was combined and compared to the same group of early disease (stages 0, I, II) of LSCC, a borderline statistically significant difference in favour of the right colon was found for grade III cancer.

## Discussion

Differences between LSCC and RSCCs have been described in many studies from different regions of the world [20, 22–26]. Globally, there is variation in the incidence of LSCC and RSCC. Considering the anatomical differences and differences in embryological origin between the two sides of the colon, colorectal cancer (CRC) presents with different clinical features. Data on the biological behaviour of CRC from studies have shown that LSCCs pursue a different clinical course compared to RSCCs [24]. These differences indicate that according to the location of the colon tumour, different methods of treatment are necessary.

In our study, we focused on differences in clinicopathological features between LSCC and RSCC in Ugandan patients. In Uganda, the median age of CRC diagnosis was 54 (IQR: 43–67) years and 38.6% were under 49 years of age. These findings are similar to those in other Sub-Saharan African countries. In Ethiopia, the overall median age was reported to be 46 years (IQR: 23–185), and in South Africa, the median age was reported to be 59 years (IQR: 14–100) [27–29]. These findings differ from the SEER demographic data of the UK (median age: 73 years) and the USA (median age: 70 years) [30, 31]. A study from the USA showed that 24.3% of CRC patients were 80 years and older, while 50% of CRC patients were 70 years and older [32].

Therefore, compared to developed high-income countries, in Sub-Saharan Africa, a higher number of patients were diagnosed at < 50 years of age with a lower median age. This difference may be explained by a difference in tumour biology or a younger population with a short life expectancy. In developed high-income countries particularly in the UK, more male patients (22,844) than female patients (18,421) develop CRC annually [33]. In other studies from Asia, male predominance has also been reported [34, 35]. Studies from Ethiopia and Kenya have shown male predominance. In Ethiopia, males constituted 62.1%, while in Kenya, males constituted 58.8% [29, 36]. A previous study from Uganda showed that out of only seventy-three CRC patients, 39 (53.4%) were women and 34 (46.6%) were male [37]. Our study showed that 49.5% were male patients and 50.5% were female patients, which is similar to findings from studies in Iran that did not observe any difference between female and male proportions in the prevalence of CRC [38–40]. We observed that female patients are diagnosed at an older age than males, and these findings are similar to studies from Western countries that showed that at the time of death, men are 4–6 years younger than women [41, 42].

There is also a major difference regarding the site of CRC between developing low-income countries and developed high-income countries. In developed high-income countries over time, a rightward shift has been observed with more frequent lesions in the proximal colon [43]. However, these developed high-income countries have recently experienced a decrease in the frequency of proximal colon tumours. Proximal lesions are possibly detected more frequently in Westernized countries than in East African countries due to the more ready availability of screening by colonoscopy [44]. Our findings show that left-sided colon tumours are more common, particularly rectal tumours, followed by sigmoid colon tumours in Uganda. This is similar to the findings from other Sub-Saharan African countries, which show a higher proportion of rectal cancers and distal colon cancers compared to findings from developed high-income countries [45]. Several Sub-Saharan African countries have shown an overwhelming predominance of the CRC rectal location [46–49], similar to our study (53%). To date, no plausible explanation has been reported; however, it may be due to a lack of screening programmes, as rectal tumours tend to present early and commonly with signs and symptoms such as rectal bleeding, rectal pain and changes in bowel habits. In Nigeria, a high prevalence of MSI-high CRC has been found, which is normally typically associated with RSCCs; however, there is still a predominance of rectal tumours [50].

Before becoming symptomatic, RSCCs grow to a large size compared to LSCCs, particularly rectal and distal colon tumours which present early with pain, haematochezia or large bowel obstruction. This may be the reason for rectal and distal colon tumours being more prevalent in developing low-income countries. In Uganda, there is no national screening programme for CRC; therefore, asymptomatic patients are rarely screened, resulting in the majority of our patients presenting with symptoms. This results in many patients presenting with advanced-stage disease, which is in keeping with findings from our study, which showed a predominance of CRC tumours presenting with either stage III or stage IV disease.

Our study has found several new findings in our Ugandan population. There was a tendency for female patients to present at an older age in RSCCs. There were more female patients presenting with CRC in the 70–79-year-old age group. Compared to older patients (≥ 46 years), many younger patients (≤ 45 years) presented with transverse colon tumours, which are RSCCs. In developed high-income countries, younger patients are more likely to present with hereditary nonpolyposis colorectal cancer (HNPCC) in RSCCs [51]. Younger patients with a family history of CRC are likely to have RSCCs [52]. However, there was otherwise no difference in the distribution of colonic tumours between male and female patients. In keeping with findings from developed high-income countries, RSCCs were more commonly detected at an advanced stage [53, 54]. These tumours present clinically with microcytic anaemia and an abdominal mass.

Our study showed a tendency for both RSCCs and LSCCs to present with an advanced stage due to increased tumour invasion in the bowel wall and increased lymph node involvement. These findings are in keeping with findings from developed high-income countries [53–55]. This advanced stage presentation may be due to a lack of national screening programmes that detect CRC at an early stage. However, in RSCCs, these findings may be the result of a long time from the initiation of carcinogenesis to the diagnosis of the tumour. In this study, a limitation was the fact that a radiological stage was used to compare the lymph node status of RSCC compared to LSCC. Determining the average number of involved lymph nodes harvested during the operation and hence determining the pathological stage would provide more accurate staging information than a radiological stage. For proper tumour staging, a minimum number of twelve lymph nodes examined in the colorectal specimen are required [53]. It has been shown that the number of harvested lymph nodes also depends on the quality of surgery and pathological examination [54, 55].

Regarding the histopathological subtypes, classical adenocarcinoma (AC) constituted 83.9% of CRC in our study. This high rate of classical adenocarcinoma (AC) is similar to the high percentages found in other studies from the West and Asia [39, 56, 57]. In the present study, the proportion of the mucinous adenocarcinoma (MAC) histopathological subtype (15.8%) was higher than the findings reported in other studies [58, 59]. Compared to American populations, mucinous adenocarcinomas (MAC) have been found to be more common in Ugandans (15.8%) versus 5.4% in Black Americans and 6.8% in White Americans.

Signet ring colorectal carcinoma (SRCC) is an aggressive histopathological subtype of CRC and showed a higher proportion of 3.9% in Ugandan patients compared to other studies from Western developed countries (< 1%) [58, 59]. This histopathological subtype was equally distributed between left-sided and right-sided CRC. Similar to another study from Nigeria, the rate of SRCC in our study was 3.9% of all adenocarcinomas and was greater than the 1.2% in the black populations of the USA and White populations [60, 61]. The high prevalence of SRCC in Ugandan patients may also partly explain the younger age of presentation of CRC compared to high-income countries [62]. SRCC generally tends to present at a younger age and is biologically more aggressive. SRCC and MAC are histopathological subtypes that are associated with frequent metastasis to the liver and peritoneum at presentation and are more difficult to diagnose, resulting in a poor prognosis [63, 64]. The high mortality of CRC in Uganda may partly be due to the higher prevalence of SRCC and MAC.

Basic clinicopathological characteristics were different between LSCC and RSCC patients. The MAC histopathological subtype had a tendency to be more common in RSCCs. This finding is in keeping with observations from other studies carried out in other regions of the world [65–68].

Compared to LSCCs, we found that advanced pT and pN stages, moderately and poorly differentiated (G2 + G3) tumours and lymphovascular invasion were more commonly found in RSCCs. Stage III and grade III tumours were more commonly seen in RSCCs (21.3%) than in LSCCs (11.7%). Although many LSCCs presented at a late stage, many were grade I lesions in contrast to late-stage RSCCs (28% versus 8.5%). These poor prognostic findings from Ugandan patients are also in keeping with findings from Western developed high-income countries [65–68].

Both in terms of grade and stage, the explanation for the worse pathological profile of RSCCs is controversial [69–71]. Although the pathological difference may not confirm this theory [11, 71], an advanced stage of presentation with RSCCs may be due to decreased screening colonoscopy [72]. In Uganda, national screening guidelines are unavailable, and therefore, the lack of faecal occult blood testing and screening colonoscopy has also resulted in many LSCC patients presenting with advanced-stage CRC in our population.

In recent decades, a growing number of researchers have focused on determining the molecular pathways leading to both LSCCs and RSCCs. In RSCCs, high microsatellite instability (MSI-H), which is the characteristic pathway in HNPCC syndrome, is characterized by a local lymphocyte reaction [73–78]. In RSCC carcinogenesis, the CpG methylation island phenotype (CIMP) and MSI-H are implicated. In LSCC carcinogenesis, the chromosomal instability pathway (CIN) and loss of heterozygosity, which correspond to mutations in the K-ras and p53 genes, are responsible [79–87].

We have described the clinicopathological features of LSCC and RSCC in Ugandan patients and have found distinct pathological differences that make them two disease entities. Compared to other regions of the world, our findings are similar. However, a higher proportion of aggressive histopathological subtypes of colorectal adenocarcinoma in particular MAC and SRCC together with an advanced stage of presentation even with LSCCs compared to Western developed high-income countries has been found in our indigenous East African population. The advanced stage at presentation irrespective of the location of the colon tumour may be primarily due to a lack of national screening guidelines in Uganda.

## Conclusions

We found that RSCC tends to be different from LSCC in terms of clinicopathological features and histology. A higher proportion of SRCC and MAC has been found in Uganda than in Western developed high-income countries, which may partly account for our high mortality. While SRCC tends to be normally distributed in the colorectum, there is a tendency for MAC to be associated with RSCC. RSCCs are more aggressive, as they are associated with a higher grade and lymphovascular invasion compared to LSCCs in our Ugandan population. However, the majority of both LSCC and RSCC patients presented with an advanced stage; therefore, national screening programmes for the early detection of CRC should be implemented to reduce mortality in our indigenous East African population.

## Limitations of study

The main limitations of this study included the following:

1. Hospital-based studies may not be representative of the entire population; however, in this study, there was a fair representation of participants from all four major regions of the country, giving a high level of confidence about the generalizability of the findings in this study.
2. To overcome the influence of antigen degradation of archival material, a high standard of laboratory testing was followed together with the maintenance of a short period of storage of specimens.
3. Another limitation was an underestimation of the stage of CRC. Staging of CRC in the years 2008–2018 mostly involved a plain chest X-ray and abdominal ultrasound scanning, with some having a CT abdomen and pelvis. In low-income developing countries such as ours, CT scanning is also largely inaccessible for many patients, especially those from rural parts of the country. Therefore, the CRC stage at diagnosis was likely to be under-assessed with inadequate high-precision staging capacity. Another reason for underestimating the CRC TNM stage in this study is that the stage was radiological at diagnosis and not pathological. The lymph node assessment in this study was also radiological and not pathological; hence, this could have underestimated the extent of lymph node involvement between right-sided and left-sided colon cancers.
4. Rectal tumours were classified as LSCCs; however, their biological behaviour is distinct from that of both LSCC and RSCCs. Rectal tumours have higher rates of Her2/neu amplification and T0P01 expression than RSCC and LSCCs. Compared to stage II and III colon tumours, similar stage rectal tumours receive neoadjuvant and adjuvant radiotherapy. Rectal tumours should therefore be considered a separate disease entity from colonic tumours.

## Supplementary Information

**Additional file 1.**

## Data Availability

The dataset generated and/or analysed during this PhD study is available as an Excel supplementary file titled: ‘Dataset for cross-sectional study’.

## Abbreviations

CRC: Colorectal cancer
RSCC: Right-sided colorectal cancer
LSCC: Left-sided colorectal cancer
AC: Classical adenocarcinoma
MAC: Mucinous adenocarcinoma
SRCC: Signet ring cell colorectal carcinoma
LVI: Lymphovascular invasion
